# Supplementary material for: Dynalign II: common secondary structure prediction for RNA homologs with domain insertions
Source: Nucleic Acids Res. 2014 Nov 21;42(22):13939–48. doi: 10.1093/nar/gku1172 (PMC4267632; doi:10.1093/nar/gku1172)
Supplement: SUPPLEMENTARY DATA [file supp_gku1172_nar-02021-z-2014-File012.zip › manual/RNA_class/html/class_dynalign__object.html]

RNAstructure Classes: Dynalign\_object Class Reference

- Main Page
- Classes
- Files

- Class List
- Class Hierarchy
- Class Members

# Dynalign\_object Class Reference

Dynalign\_object Class.
More...

`#include <Dynalign_object.h>`

Inherits TwoRNA.

List of all members.

|  |  |
| --- | --- |
|  |  |
| Public Member Functions | |
|  | Dynalign\_object () |
|  | Dynalign\_object (const char sequence1[], const char sequence2[], const bool IsRNA=true) |
|  | Dynalign\_object (const char filename1[], const int type1, const char filename2[], const int type2, const bool IsRNA=true) |
|  | Constructor. |
|  | Dynalign\_object (const char filename[]) |
|  | Constructor. |
|  | Dynalign\_object (const char \*filename, const short maxtrace, const short bpwin, const short awin, const short percent) |
| int | Dynalign (const short int maxtrace, const short int bpwin, const short int awin, const short int percent, const short int imaxseparation=-99, const float gap=0.4, const bool singleinsert=true, const char savefile[]=NULL, const bool optimalonly=false, const short int singlefold\_subopt\_percent=30, const bool local=false, const short int numProcessors=1, const int maxpairs=-1) |
|  | Predict the lowest free energy structure common to two sequences and suboptimal solutions with the Dynalign algorithm. |
| void | WriteAlignment (const char filename[]) |
|  | Write the alignment to disk. |
| int | ForceAlignment (const int i, const int k) |
|  | Force an alignment during a Dynalign calculation). |
| int | GetForcedAlignment (const int i, const int seq) |
|  | Get an alignment constraint. |
| int | ReadAlignmentConstraints (const char filename[]) |
|  | Read alignment constraints from disk. |
| int | Templatefromct (const char ctfilename[]) |
|  | Read a ct file to determine what pairs will be allowed for sequence 1 in a subsequent dynalign calculation. |
| int | Templatefromdsv (const char dsvfilename[], const float maxdsvchange) |
| double | GetBestPairEnergy (const int sequence, const int i, const int j) |
|  | Report the best energy for pair i-j from sequence number sequence (1 or 2). |
| double | GetLowestEnergy () |
|  | Report the lowest total free energy change from a Dynalign calculation. |
| char \* | GetErrorMessage (const int error) |
|  | Return error messages based on code from GetErrorCode and other error codes. |
| void | SetProgress (TProgressDialog &Progress) |
| void | StopProgress () |
|  | ~Dynalign\_object () |
| Private Member Functions | |
| void | CommonConstructor () |
| void | AllocateForceAlign () |
| void | storetemplatefilename (const char \*name) |
| Private Attributes | |
| short \*\* | align |
| short \*\* | forcealign |
| bool | dsv\_templated |
| bool | ct\_templated |
| char \* | templatefilename |
| float | MAXDSV |
| int | modificationflag |
| dynalignarray \* | w |
| dynalignarray \* | vmod |
| varray \* | v |
| wendarray \* | w5 |
| wendarray \* | w3 |
| short \* | lowend |
| short \* | highend |
| datatable \* | data |
| short | gap |
| short | lowest |
| int | Maxtrace |
| bool | savefileread |
| double \*\*\* | array |

---

## Detailed Description

Dynalign\_object Class.

The Dynalign\_object class provides an entry point for the Dynalign algorithm. The class is inherited from the TwoRNA class, which itself contains two instances to the class RNA. 

---

## Constructor & Destructor Documentation

|  |  |  |  |  |
| --- | --- | --- | --- | --- |
| Dynalign\_object::Dynalign\_object | ( |  | ) |  |

Constructor This is a default constructor that calls the TwoRNA default constructor.

|  |  |  |  |  |
| --- | --- | --- | --- | --- |
| Dynalign\_object::Dynalign\_object | ( | const char | *sequence1*[], |  |
|  |  | const char | *sequence2*[], |  |
|  |  | const bool | *IsRNA* = `true` |  |
|  | ) |  |  |  |

This constuctor is available to reach the TwoRNA constructors, from which this class is inherited. This constructor uses two cstrings to provide sequences. IsRNA is true for RNA folding and flase for DNA. Constructor This constuctor is available to reach the TwoRNA constructors, from which this class is inherited. This constructor uses two cstrings to provide sequences. IsRNA is true for RNA folding and false for DNA. Input sequences should contain A,C,G,T,U,a,c,g,t,u,x,X. Capitalization makes no difference. T=t=u=U. If IsRNA is true, the backbone is RNA, so U is assumed. If IsRNA is false, the backbone is DNA, so T is assumed. x=X= nucleotide that neither stacks nor pairs. For now, any unknown nuc is considered 'X'. Both sequences are passed to underlying RNA classes for each sequence.

**Parameters:**
:   |  |  |  |
    | --- | --- | --- |
    |  | *sequence1* | is a NULL terminated c string for sequence 1. |
    |  | *sequence2* | is a NULL terminated c string for sequence 2. |
    |  | *IsRNA* | is a bool that indicates whether these sequences are RNA or DNA. true=RNA. false=DNA. Default is true. Both sequences must have the same backbone. |

|  |  |  |  |  |
| --- | --- | --- | --- | --- |
| Dynalign\_object::Dynalign\_object | ( | const char | *filename1*[], |  |
|  |  | const int | *type1*, |  |
|  |  | const char | *filename2*[], |  |
|  |  | const int | *type2*, |  |
|  |  | const bool | *IsRNA* = `true` |  |
|  | ) |  |  |  |

Constructor.

This constuctor is available to reach the TwoRNA constructors, from which this class is inherited. This constructor uses two ctsirngs as filenames, accompanied by integers to set the file type. IsRNA is true for RNA folding and false for DNA. The existing files, specified by filenames, can either be a ct file, a sequence, or an RNAstructure save file. Therefore, the user provides a flag for the file type: type = 1 => .ct file, type = 2 => .seq file, type = 3 => partition function save (.pfs) file, type = 4 => folding save file (.sav). The file opening is performed by the constructors for the RNA classes that underlie each sequence. This constructor generates internal error codes that can be accessed by GetErrorCode() after the constructor is called. 0 = no error. The errorcode can be resolved to a c string using GetErrorMessage. Note that the contructor needs to be explicitly told, via IsRNA, what the backbone is because files do not store this information. Note also that save files explicitly store the thermodynamic parameters, therefore changing the backbone type as compaared to the original calculation will not change structure predictions.

**Parameters:**
:   |  |  |  |
    | --- | --- | --- |
    |  | *filename1* | is a null terminated c string and refers to sequence 1. |
    |  | *filename2* | is a null terminated c string and refers to sequence 2. |
    |  | *type1* | is an integer that indicates the file type for sequence 1. |
    |  | *type2* | is an integer that indicates the file type for sequence 2. |
    |  | *IsRNA* | is a bool that indicates whether these sequences are RNA or DNA. true=RNA. false=DNA. Default is true. Only one backbone is allowed for both sequences. |

|  |  |  |  |  |  |
| --- | --- | --- | --- | --- | --- |
| Dynalign\_object::Dynalign\_object | ( | const char | *filename*[] | ) |  |

Constructor.

This constructor allows the user to read a dynalign save file (.dsv) to get base pairing information. This constructor generates internal error codes that can be accessed by GetErrorCode() after the constructor is called. 0 = no error. The errorcode can be resolved to a c string using GetErrorMessage.

**Parameters:**
:   |  |  |  |
    | --- | --- | --- |
    |  | *filename[]* | is a cstring that indicates the filename of a .dsv file. |

|  |  |  |  |  |
| --- | --- | --- | --- | --- |
| Dynalign\_object::Dynalign\_object | ( | const char \* | *filename*, |  |
|  |  | const short | *maxtrace*, |  |
|  |  | const short | *bpwin*, |  |
|  |  | const short | *awin*, |  |
|  |  | const short | *percent* |  |
|  | ) |  |  |  |

Constructor This constructor is used to perform Dynaligh refolding. This does not allow any changes in constraints, but does allow the creation of different set of suboptimal structures. This constructor generates internal error codes that can be accessed by GetErrorCode() after the constructor is called. 0 = no error. The errorcode can be resolved to a c string using GetErrorMessage.

**Parameters:**
:   |  |  |  |
    | --- | --- | --- |
    |  | *filename* | is the name of a Dynalign save file name (.dsv). |
    |  | *maxtrace* | is the maximum number of common structures to be determined. |
    |  | *bpwin* | the the base pair window parameter, where 0 allows the structures to have similar pairs and larger windows make the structures more diverse. |
    |  | *awin* | is the alignment window parameter, where 0 allows the alignments to be similar and larger values make the alignments more diverse. |
    |  | *percent* | is the maximum percent difference in total folding free energy change above the lowest for suboptimal common structures. |

|  |  |  |  |  |
| --- | --- | --- | --- | --- |
| Dynalign\_object::~Dynalign\_object | ( |  | ) |  |

---

## Member Function Documentation

|  |  |  |  |  |
| --- | --- | --- | --- | --- |
| void Dynalign\_object::AllocateForceAlign | ( |  | ) | `[private]` |

|  |  |  |  |  |
| --- | --- | --- | --- | --- |
| void Dynalign\_object::CommonConstructor | ( |  | ) | `[private]` |

|  |  |  |  |  |
| --- | --- | --- | --- | --- |
| int Dynalign\_object::Dynalign | ( | const short int | *maxtrace*, |  |
|  |  | const short int | *bpwin*, |  |
|  |  | const short int | *awin*, |  |
|  |  | const short int | *percent*, |  |
|  |  | const short int | *imaxseparation* = `-99`, |  |
|  |  | const float | *gap* = `0.4`, |  |
|  |  | const bool | *singleinsert* = `true`, |  |
|  |  | const char | *savefile*[] = `NULL`, |  |
|  |  | const bool | *optimalonly* = `false`, |  |
|  |  | const short int | *singlefold\_subopt\_percent* = `30`, |  |
|  |  | const bool | *local* = `false`, |  |
|  |  | const short int | *numProcessors* = `1`, |  |
|  |  | const int | *maxpairs* = `-1` |  |
|  | ) |  |  |  |

Predict the lowest free energy structure common to two sequences and suboptimal solutions with the Dynalign algorithm.

In case of error, the function returns a non-zero that can be parsed by GetErrorMessage() or GetErrorMessageString().

**Parameters:**
:   |  |  |  |
    | --- | --- | --- |
    |  | *maxtrace* | is the maximum number of common structures to be determined. |
    |  | *bpwin* | the the base pair window parameter, where 0 allows the structures to have similar pairs and larger windows make the structures more diverse. |
    |  | *awin* | is the alignment window parameter, where 0 allows the alignments to be similar and larger values make the alignments more diverse. |
    |  | *percent* | is the maximum percent difference in total folding free energy change above the lowest for suboptimal common structures. |
    |  | *imaxseparation* | is the maximum separation between aligned nucleotides. Values >= 0 are the traditional parameter, those below zero trigger the HMM alignment method, which is now prefered. |
    |  | *gap* | is the cost of adding gap nucleotides in the alignment in kcal/mol. |
    |  | *singleinsert* | is whether single basepair inserts are allowed in one sequence vs the other. |
    |  | *savefile* | is c-string with the name of a dynalign savefile (\*.dsv) to be created. |
    |  | *optimalonly* | can be used to turn on a calculation of only the energy (when true) and not the structures. |
    |  | *singlefold\_subopt\_percent* | is the maximum % difference of folding energy above the lowest free energy structure for pairs in single sequence folding that will be allowed in the dynalign calculation. |
    |  | *local* | is whether Dynalign is being run in local (true) or global mode (false). |
    |  | *numProcessors* | is the number of processors to use for the calculation. This requires a compilation for SMP. |
    |  | *maxpairs* | is under development for multiple sequence folding. Use -1 (default) for now. |

**Returns:**
:   An int that indicates an error code (0 = no error, non-zero = error occurred).

|  |  |  |  |  |
| --- | --- | --- | --- | --- |
| int Dynalign\_object::ForceAlignment | ( | const int | *i*, |  |
|  |  | const int | *k* |  |
|  | ) |  |  |  |

Force an alignment during a Dynalign calculation).

Nucleotide i from sequence 1 will be aligned to nucleotide k in sequence 2 in subsequent Dynalign calculation. The function returns 0 with no error and a non-zero otherwise that can be parsed by GetErrorMessage() or GetErrorMessageString().

**Parameters:**
:   |  |  |  |
    | --- | --- | --- |
    |  | *i* | is the index of nucleotide from sequence 1. |
    |  | *k* | is the index of nucleotide from sequence 2. |

**Returns:**
:   An integer that indicates an error code (0 = no error, 100 = nucleotide i out of range, 101 = nucleotide k out of range).

|  |  |  |  |  |
| --- | --- | --- | --- | --- |
| double Dynalign\_object::GetBestPairEnergy | ( | const int | *sequence*, |  |
|  |  | const int | *i*, |  |
|  |  | const int | *j* |  |
|  | ) |  |  |  |

Report the best energy for pair i-j from sequence number sequence (1 or 2).

This function reports the lowest ffolding free energy for any pairs between i-j in sequence number sequence (1 or 2). This requires a search over all possible pairs in the second sequence. NOTE: This function ONLY works after reading a Dynalign save file (.dsv) using the constructor. This is because the Dynalign energies are not normally stored after calling Dynalign. This function generates internal error codes that can be accessed by GetErrorCode() after the constructor is called. 0 = no error, 107 = Data not available, 108 = nucleotide out of range. The errorcode can be resolved to a c string using GetErrorMessage.

**Parameters:**
:   |  |  |  |
    | --- | --- | --- |
    |  | *sequence* | is an integer indicating the sequence # (must be 1 or 2). |
    |  | *i* | is the 5' nucleotide in a pair. |
    |  | *j* | is the 3' nucleotide in a pair. |

**Returns:**
:   A double that gives an energy in kcal/mol.

|  |  |  |  |  |  |
| --- | --- | --- | --- | --- | --- |
| char \* Dynalign\_object::GetErrorMessage | ( | const int | *error* | ) |  |

Return error messages based on code from GetErrorCode and other error codes.

0 = no error 100-999 = Error associated with Dynalign, to be handled here. >=1000 = Errors for underlying sequence, get message from TwoRNA base class. Current errors handled here are: 100 "Nucleotide from sequence 1 is out of range.\n"; 101 "Nucleotide from sequence 2 is out of range.\n"; 102 "Alignment constraint file not found.\n"; 103 "Error reading alignment constraint file.\n"; 104 "CT file not found.\n"; 105 "A template has already been specified; only one is allowed.\n"; 106 "DSV file not found.\n"; 107 "Data not available to calculate energy.\n" 108 "Nucleotide out of range.\n"; 109 "Value of maxpairs is too large to be achievable.\n" 110 "Error reading thermodynamic parameters."

**Parameters:**
:   |  |  |  |
    | --- | --- | --- |
    |  | *error* | is the integer error code provided by GetErrorCode() or by a call to a function that returns an error. |

**Returns:**
:   A pointer to a c string that provides an error message or from other functions that return integer error codes.

Reimplemented from TwoRNA.

|  |  |  |  |  |
| --- | --- | --- | --- | --- |
| int Dynalign\_object::GetForcedAlignment | ( | const int | *i*, |  |
|  |  | const int | *seq* |  |
|  | ) |  |  |  |

Get an alignment constraint.

**Parameters:**
:   |  |  |  |
    | --- | --- | --- |
    |  | *i* | is the nucleotide number. |
    |  | *seq* | is the sequence (1 or 2) from which i is derived. |

**Returns:**
:   An integer that indicates the nucleotide to which i is forced to be aligned, where 0 indicates no alignment.

|  |  |  |  |  |
| --- | --- | --- | --- | --- |
| double Dynalign\_object::GetLowestEnergy | ( |  | ) |  |

Report the lowest total free energy change from a Dynalign calculation.

NOTE: This function ONLY works after reading a Dynalign save file (.dsv) using the constructor. This is because the Dynalign energies are not normally stored after calling Dynalign. This function generates internal error codes that can be accessed by GetErrorCode() after the constructor is called. 0 = no error, 107 = Data not available, 108 = nucleotide out of range. The errorcode can be resolved to a c string using GetErrorMessage.

**Returns:**
:   a double that gives an energy in kcal/mol.

|  |  |  |  |  |  |
| --- | --- | --- | --- | --- | --- |
| int Dynalign\_object::ReadAlignmentConstraints | ( | const char | *filename*[] | ) |  |

Read alignment constraints from disk.

The file format is: i1 k1 i2 k2 -1 -1 Where each line gives a aligned pair (i from sequence 1 and k from sequence 2). The file terminates with -1 -1 to indicate the file end. The function returns 0 with no error and a non-zero otherwise that can be parsed by GetErrorMessage() or GetErrorMessageString().

**Parameters:**
:   |  |  |  |
    | --- | --- | --- |
    |  | *filename* | is a c string that is the file name to be read. |

**Returns:**
:   An integer that indicates an error code (0 = no error, 102 = file not found, 103 = error reading constraint file).

|  |  |  |  |  |  |
| --- | --- | --- | --- | --- | --- |
| void Dynalign\_object::SetProgress | ( | TProgressDialog & | *Progress* | ) |  |

Provide a TProgressDialog for following calculation progress. A TProgressDialog class has a public function void update(int percent) that indicates the progress of a long calculation.

**Parameters:**
:   |  |  |  |
    | --- | --- | --- |
    |  | *Progress* | is a TProgressDialog class. |

|  |  |  |  |  |
| --- | --- | --- | --- | --- |
| void Dynalign\_object::StopProgress | ( |  | ) |  |

Provide a means to stop using a TProgressDialog. StopProgress tells the RNA class to no longer follow progress. This should be called if the TProgressDialog is deleted, so that this class does not make reference to it.

|  |  |  |  |  |  |
| --- | --- | --- | --- | --- | --- |
| void Dynalign\_object::storetemplatefilename | ( | const char \* | *name* | ) | `[private]` |

|  |  |  |  |  |  |
| --- | --- | --- | --- | --- | --- |
| int Dynalign\_object::Templatefromct | ( | const char | *ctfilename*[] | ) |  |

Read a ct file to determine what pairs will be allowed for sequence 1 in a subsequent dynalign calculation.

This results in all pairs but those in the ct being disallowed.

**Parameters:**
:   |  |  |  |
    | --- | --- | --- |
    |  | *ctfilename* | is the name of the ct file to be read to provide the template. |

**Returns:**
:   An integer that indicates an error code (0=no error, 104=file not found, 105=template is already specified)

|  |  |  |  |  |
| --- | --- | --- | --- | --- |
| int Dynalign\_object::Templatefromdsv | ( | const char | *dsvfilename*[], |  |
|  |  | const float | *maxdsvchange* |  |
|  | ) |  |  |  |

This reads a dsv file and only allows pairs with folding free energy change between the lowest and lowest + maxdsvchange in a subsequent dynalign calculation.

**Parameters:**
:   |  |  |  |
    | --- | --- | --- |
    |  | *dsvfilename* | is the name of the ct file to be read to provide the template. |
    |  | *maxdsvchange* | in a float that gives a percent difference in free energy above the lowest free energy change. |

**Returns:**
:   An integer that indicates an error code (0=no error, 106=file not found, 105=template is already specified)

|  |  |  |  |  |  |
| --- | --- | --- | --- | --- | --- |
| void Dynalign\_object::WriteAlignment | ( | const char | *filename*[] | ) |  |

Write the alignment to disk.

This function should be called after loading a dynalign save file or after a dynalign calculation has been performed. This function generates no error flag. Nothing can go wrong...

**Parameters:**
:   |  |  |  |
    | --- | --- | --- |
    |  | *filename* | is the file to which the alignment should be written. |

---

## Member Data Documentation

short\*\* Dynalign\_object::align `[private]`

double\*\*\* Dynalign\_object::array `[private]`

bool Dynalign\_object::ct\_templated `[private]`

datatable\* Dynalign\_object::data `[private]`

bool Dynalign\_object::dsv\_templated `[private]`

short\*\* Dynalign\_object::forcealign `[private]`

short Dynalign\_object::gap `[private]`

short \* Dynalign\_object::highend `[private]`

short\* Dynalign\_object::lowend `[private]`

short Dynalign\_object::lowest `[private]`

float Dynalign\_object::MAXDSV `[private]`

int Dynalign\_object::Maxtrace `[private]`

int Dynalign\_object::modificationflag `[private]`

bool Dynalign\_object::savefileread `[private]`

char\* Dynalign\_object::templatefilename `[private]`

varray\* Dynalign\_object::v `[private]`

dynalignarray \* Dynalign\_object::vmod `[private]`

dynalignarray\* Dynalign\_object::w `[private]`

wendarray \* Dynalign\_object::w3 `[private]`

wendarray\* Dynalign\_object::w5 `[private]`

---

The documentation for this class was generated from the following files:

- F:/RNAstructure-co-from-repository/RNAstructure/RNA\_class/Dynalign\_object.h- F:/RNAstructure-co-from-repository/RNAstructure/RNA\_class/Dynalign\_object.cpp

---

Generated on Tue Jul 30 10:52:29 2013 for RNAstructure Classes by 
 1.5.7.1
